# Fires and the Architects

**Published:** 1902-06-28

**Authors:** 


### FIRES AND THE ARCHITECTS.

J. COMPTON MERRYWEATHER, of Whitehall Court, S.W., writes to point out that unless the position of external iron fire-escape ladders be carefully arranged they may prove useless should fire break out on a lower floor and the flames from the windows sweep across them. At the North Eastern Hospital at Homerton, he says, "they have chute fire-escapes, and these canvas tubes certainly offer the quickest and safest mode of exit from a burning building. They are also inexpensive, and may thus be adopted by many hospitals whose funds are low."

